# Facet-selective anisotropic growth of 1D calcite nanowhiskers via Mg/Zn/SO₄ additive-assisted carbonation

**DOI:** 10.1038/s41598-026-52737-8

**Published:** 2026-05-09

**Authors:** Seungyeol Lee

**Affiliations:** Department of Earth and Environmental Sciences, Chungbuk National University, S1-6 608, 1 Chungdae-ro, Seowon-gu, Cheongju, Chungbuk 28644 Republic of Korea

**Keywords:** Calcium carbonate, Calcite whisker, Additive-assisted carbonation, Facet-selective growth, Crystal morphology, Chemistry, Materials science, Nanoscience and technology

## Abstract

Achieving highly anisotropic, whisker-like calcium carbonate (CaCO₃) architectures while retaining the thermodynamically stable calcite phase remains a significant challenge. Here, we present a staged additive-assisted carbonation strategy employing Mg, Zn, and SO₄ species to produce sub-micrometer calcite nanorods and whiskers. Powder X-ray diffraction identifies the product as calcite without detectable metastable polymorphs, and scanning electron microscopy reveals highly porous, cauliflower-like secondary agglomerates. Transmission electron microscopy directly visualizes the embedded one-dimensional primary nano-units (lengths 393–406 nm; aspect ratios 4–7), and indexed lattice fringes establish their identity as calcite. N₂ physisorption further indicates a mesopore-dominated architecture with a specific surface area of 9.58 m²/g. On the basis of these multiscale observations and the established crystal-chemical behavior of Mg²⁺, Zn²⁺, and SO₄²⁻ at carbonate surfaces, we propose a facet-selective growth model in which synergistic kinetic inhibition by Mg and SO₄ suppresses lateral thickening while Zn-mediated surface interactions are inferred to promote anisotropic elongation along the crystallographic *c*-axis. These findings provide mechanistic insights and a scalable pathway for engineering one-dimensional calcite morphologies for advanced structural and environmental applications.

## Introduction

Calcium carbonate (CaCO₃) is one of the most abundant and cost-effective inorganic minerals, finding widespread use as a functional filler in polymers, elastomers, coatings, and paper products^1^. The physicochemical performance of CaCO₃ in these composite systems is governed not solely by its bulk composition but rather by particle size, morphological habit, and interfacial area. Accordingly, nanostructuring and surface engineering of CaCO₃ have attracted considerable attention as effective strategies for enhancing mechanical reinforcement and enabling surface-driven functionalities. Recent progress in the controlled synthesis of nanoscale CaCO₃ further highlights the importance of tailoring both crystal morphology and accessible surface area to meet the stringent requirements of advanced structural and environmental applications^2^.

Among various morphological designs, one-dimensional (1D) nanostructures such as whiskers and nanorods offer distinct advantages. Within composite matrices, these 1D CaCO₃ architectures can form percolated networks and promote mechanical interlocking, thereby substantially improving stress transfer and crack-bridging performance. Nevertheless, the synthesis of whisker-like CaCO₃ has largely been confined to metastable polymorphs, most notably aragonite^3^. Achieving anisotropic, whisker-like growth in calcite—the thermodynamically most stable CaCO₃ polymorph under ambient conditions—remains a formidable challenge^4,5^. This difficulty stems from the intrinsic crystallization habit of calcite, which strongly favors rhombohedral morphologies that minimize surface energy through the expression of highly stable {104} facets^6^. Overcoming this thermodynamic preference demands precise kinetic intervention to selectively inhibit these low-energy facets during nucleation and crystal growth.

Within both classical and nonclassical frameworks, inorganic additives are now recognized as central regulators of CaCO₃ crystallization, modulating polymorph selection, nucleation kinetics, and morphological evolution by altering prenucleation cluster speciation, the stability of amorphous calcium carbonate (ACC), and the thermodynamics of step propagation on growing crystal faces^2,7,8^. Magnesium plays a particularly multifaceted role in this regard: it stabilizes Mg-incorporated ACC precursors whose subsequent transformation can be directed toward calcite, aragonite, or high-Mg calcite depending on solution chemistry and reaction temperature^9,10^, while simultaneously retarding calcite step propagation through dehydration-limited incorporation at acute step edges, as established by direct atomic-force-microscopy measurements^11^. Sulfate ions further modify the interfacial water structure and surface free energy of calcite, often acting cooperatively with Mg²⁺ to alter the relative growth rates of crystallographically distinct faces^6,12^. Zinc, by contrast, has received comparatively less attention in CaCO₃ systems but is known to bind preferentially to structurally distinct termination sites of carbonates and to influence facet stability through inner-sphere surface complexation^13,14^. Despite these advances, prior additive-mediated studies have predominantly addressed polymorph selection (calcite vs. aragonite) or amorphous-precursor-mediated transformations, with comparatively little attention to the rational engineering of one-dimensional, whisker-like calcite morphologies. The present work extends this body of literature by demonstrating that a tailored Mg/Zn/SO₄ co-additive system can override the intrinsic {104} growth habit of calcite and direct anisotropic elongation along the *c*-axis, thereby providing a route to high-aspect-ratio calcite that is mechanistically distinct from ACC-precursor or biomolecule-templated strategies.

To address this challenge, we present a staged, additive-assisted carbonation strategy that employs a tailored combination of Mg, Zn, and SO₄ species^11–13^ to produce sub-micrometer calcite nanorods and whiskers embedded within highly porous secondary agglomerates. Through the systematic integration of phase and thermal analyses, textural characterization, and high-resolution electron microscopy, we elucidate the morphological evolution of these hierarchical structures. On the basis of these observations, we propose a mechanistic growth model illustrating how synergistic, facet-selective interactions mediated by these additives can effectively override the intrinsic {104} growth habit, directing the crystallization pathway toward anisotropic elongation while preserving the thermodynamically stable calcite phase^14^.

## Materials and methods

### Synthesis procedure

High-aspect-ratio calcite nanowhiskers were synthesized via a staged, additive-assisted gas–liquid carbonation route based on Ca(OH)₂–CO₂ mineralization. The reactor system employed here was originally developed for the synthesis of ~ 100 nm particulate nano-calcium carbonate rather than whisker morphologies; detailed experimental procedures are described in our earlier report^1^. Calcium oxide (CaO, ≥ 98% purity, particle size < 75 μm; Sigma-Aldrich) was used as the calcium precursor, and high-purity CO₂ gas (99.5%; Linde Korea, Gyeonggi-do, Republic of Korea) was used as received. The carbonation reactor comprised a stainless-steel cylindrical vessel with an internal diameter of 1.5 m and a height of 2.0 m. Each batch was initiated at an initial slurry temperature of approximately 14 °C, and the temperature subsequently rose progressively to ~ 30 °C over the course of the ~ 6 h carbonation. This thermal evolution arose primarily from the exothermic nature of the gas–liquid carbonation reaction (Ca(OH)₂ + CO₂ → CaCO₃ + H₂O, ΔH°_rxn_ₓₙ≈ −113 kJ mol⁻¹), the heat of which raised the bulk temperature of the 20-kg slurry within the passively-thermostated stainless-steel vessel; no active cooling was applied. The 14–30 °C envelope was retained intentionally rather than imposing tighter isothermal control, as it lies well below the threshold at which aragonite formation becomes kinetically favored in Mg²⁺-bearing carbonation systems while still providing adequate driving force for calcite nucleation and limiting Ostwald ripening of the primary nanorods. The pH and temperature were monitored continuously throughout the process to track reaction progress.

To regulate nucleation and anisotropic growth, metal sulfate additives were introduced in a time-staggered sequence. Immediately before CO₂ injection, solid magnesium sulfate (MgSO₄; Sigma-Aldrich) was added to the Ca(OH)₂ slurry at a molar ratio of 0.01–0.03 relative to Ca(OH)₂. This initial addition served to suppress lateral growth during the early stages of crystallization, exploiting the well-established preferential binding of Mg²⁺ to acute step edges of calcite {104} faces and the consequent retardation of lateral step propagation^11^. Carbonation was then initiated by continuously injecting CO₂ through a gas ejector system to ensure direct gas–liquid contact and efficient mass transfer. The CO₂ flow rate was controlled at 100 L min⁻¹ per kilogram of Ca(OH)₂. Concurrently with CO₂ injection, solid zinc sulfate (ZnSO₄; Sigma-Aldrich) was introduced at 0.02–0.15 mol per mol of Ca(OH)₂, either alone or in combination with liquid sulfuric acid (H₂SO₄; Sigma-Aldrich). When both reagents were co-administered, the ZnSO₄-to-H₂SO₄ mass ratio was maintained between 1:1 and 10:1. This staged dosing strategy was designed to inhibit lateral facet advancement while promoting preferential elongation along the crystallographic *c*-axis. The carbonation reaction was allowed to proceed until near-complete conversion was achieved—typically requiring approximately 6 h under these conditions—or until the slurry pH decreased to approximately 6.8, at which point the CO₂ supply was terminated.

Following the reaction, the aqueous CaCO₃ slurry was transferred to a storage tank for direct liquid-phase application if desired. For powder recovery, the product slurry was dewatered and subsequently dried at room temperature under ambient laboratory conditions without external heating. The dried product was then deagglomerated and classified to yield the final nano-calcite whisker powder.

### Characterization

The crystallographic phase purity and macroscopic morphology of the synthesized calcite nanowhiskers were characterized at the Mineralogy and Mineral Resources Laboratory, Department of Earth and Environmental Sciences, Chungbuk National University (CBNU). Powder X-ray diffraction (XRD) was performed on a Rigaku MiniFlex 600 diffractometer (Tokyo, Japan) using Cu Kα radiation (λ = 1.5406 Å) at an accelerating voltage of 40 kV and an emission current of 15 mA. Diffraction patterns were collected over a 2θ range of 5–80° with a step size of 0.01° and a scan rate of 3° min⁻¹. The instrument was calibrated against a standard silicon reference material prior to measurement to minimize systematic angular deviations, and phase identification was carried out using standard reference patterns^15^. Morphological features and elemental compositions were examined by scanning electron microscopy (SEM; JEOL JSM-IT510, Tokyo, Japan) at an accelerating voltage of 10–15 kV in high-vacuum mode. The microscope was equipped with an Oxford Instruments energy-dispersive X-ray spectroscopy (EDS) detector for qualitative and semi-quantitative elemental mapping.

Nanoscale, textural, and thermal characterizations were performed at the Central Instrumentation Facility of CBNU. Nanoscale morphology and lattice fringes were examined by spherical-aberration (Cs)-corrected transmission electron microscopy (TEM; JEM-ARM200F NEOARM, JEOL Ltd., Tokyo, Japan) operating at 200 kV. Fast Fourier transform (FFT) patterns were derived from high-resolution TEM (HRTEM) images to evaluate local crystallinity and crystallographic orientation. TEM specimens were prepared by dispersing the powder in ethanol, sonicating for 10 min to reduce particle agglomeration, and drop-casting the resulting suspension onto carbon-coated lacey copper grids followed by drying under ambient conditions.

The specific surface area and pore characteristics were determined from N₂ adsorption–desorption isotherms measured at 77 K using a Micromeritics ASAP 2425 porosimetry analyzer. Several samples with a mass of ~ 0.15 g were analyzed with a 10 s equilibration interval. The Brunauer–Emmett–Teller (BET) method was used to calculate the specific surface area, while pore size distributions and pore volumes were obtained by the Barrett–Joyner–Halenda (BJH) and t-plot methods. Thermal decomposition behavior was investigated by thermogravimetric analysis (TGA) coupled with derivative thermogravimetry (DTG) using a NETZSCH TG 209F1 Libra instrument. Approximately 6.87 mg of powder was placed in an open Al₂O₃ crucible (85 µL) and heated from 30 to 1100 °C at a constant rate of 25 K min⁻¹ under a controlled dual-gas atmosphere of synthetic air (250.3 mL min⁻¹) and nitrogen (250.0 mL min⁻¹ for both purge and protective streams). Baseline correction was applied using a method-specific correction file to ensure accurate determination of decarbonation kinetics^16,17^.

## Results and discussion

### Crystallographic phase and structural integrity

**Fig. 1:**
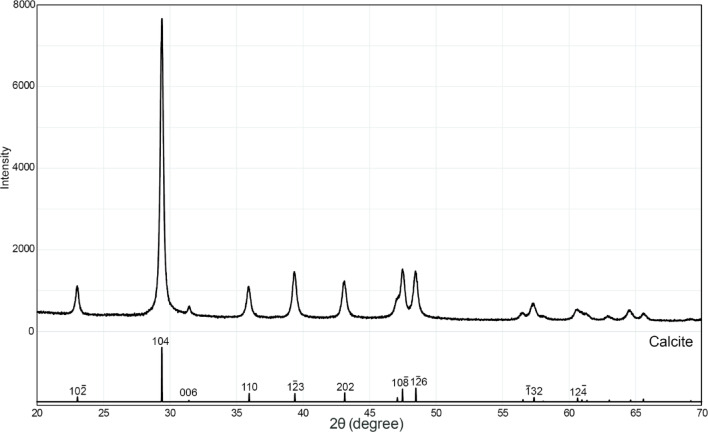
Powder X-ray diffraction (XRD) pattern of the synthesized nano-calcite sample, confirming rhombohedral calcite as the dominant crystalline phase. All identified reflections coincide with those tabulated for pure calcite, with no detectable shifts attributable to bulk-lattice substitution by Mg²⁺ or Zn²⁺. The relatively broad line widths are consistent with the nanoscale dimensions of the primary crystalline domains revealed by electron microscopy.

The powder X-ray diffraction (XRD) pattern of the synthesized product confirms rhombohedral calcite as the sole crystalline phase (Fig. 1). The dominant reflection at 2θ ≈ 29.4° corresponds to the calcite (104) plane, accompanied by a characteristic series of secondary reflections indexed to the (012), (006), (110), (113), and (202) planes^15,18^. No reflections attributable to metastable CaCO₃ polymorphs such as aragonite or vaterite were detected. This finding is particularly noteworthy because one-dimensional whisker- or needle-like morphologies in CaCO₃ are most commonly associated with the aragonite phase on both thermodynamic and kinetic grounds^3^. The complete absence of aragonite therefore indicates that the staged Mg/Zn/SO₄ additive system imposes strict kinetic control over the crystallization pathway, directing crystal growth toward high-aspect-ratio morphologies while preserving the thermodynamic stability of calcite^7^.

The diffraction line widths are relatively broad (FWHM of the (104) reflection on the order of 0.8°), in keeping with the nanoscale dimensions of the primary crystalline domains revealed by electron microscopy and with possible microstrain contributions characteristic of additive-modulated CaCO₃. We therefore base our interpretation of the additive distribution on the *angular positions* of the diffraction peaks rather than on their widths. Within the experimental angular resolution, all reflections coincide with those tabulated for pure calcite^15^; we observe no systematic peak shifts of the type that would be diagnostic of substantial Mg²⁺ or Zn²⁺ substitution into the bulk calcite lattice or of the formation of solid-solution phases such as dolomite or zincian calcite^7^.

### Thermal decomposition behavior

**Fig. 2:**
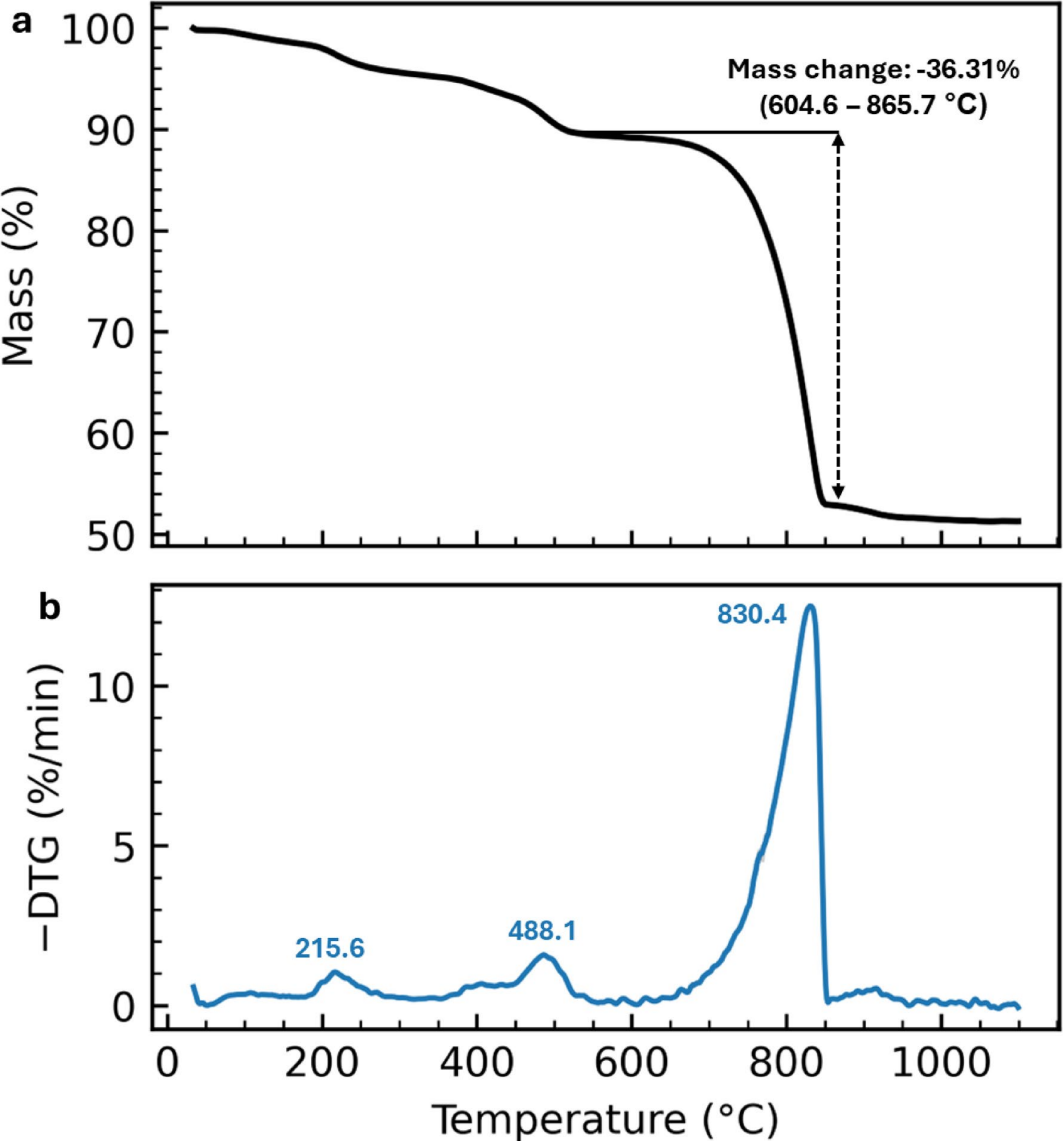
Thermogravimetric (TG) and derivative thermogravimetric (DTG) profiles of the nano-calcite sample, recorded from 30 to 1100 °C at a constant heating rate of 25 K min⁻¹. The dominant DTG peak at ~ 830–850 °C corresponds to the endothermic decarbonation of CaCO₃ to CaO and CO₂.

Thermogravimetric analysis reveals a multi-step mass-loss profile over the temperature range of 30–1100 °C (Fig. 2). A minor mass decrease is observed below 200 °C, followed by a gradual decline between 200 and 600 °C, before a dominant decomposition event occurs between 740 and 880 °C. The corresponding derivative thermogravimetry (DTG) curve exhibits a pronounced peak centered at approximately 830–850 °C, consistent with the endothermic decarbonation of CaCO₃ to CaO and CO₂^17^. The relatively elevated peak temperature can be attributed to the rapid heating rate employed (25 K min⁻¹), which is a well-documented kinetic factor that shifts thermal events to higher temperatures owing to thermal lag and heat-transfer limitations^19^. The residual mass at 1100 °C is approximately 51–52 wt%. The broad DTG features observed between 200 and 600 °C are ascribed to the desorption of strongly bound molecular species, structural water, or dehydroxylation of residual hydroxide phases, events that typically occur near 450–550 °C in highly porous calcium-bearing systems^1,20^. Importantly, the high thermal stability demonstrated here is consistent with the XRD results, confirming that the nanoscale one-dimensional morphology and elevated surface area do not compromise the intrinsic thermal resilience of the calcite lattice.

### Hierarchical morphology across length scales

**Fig. 3:**
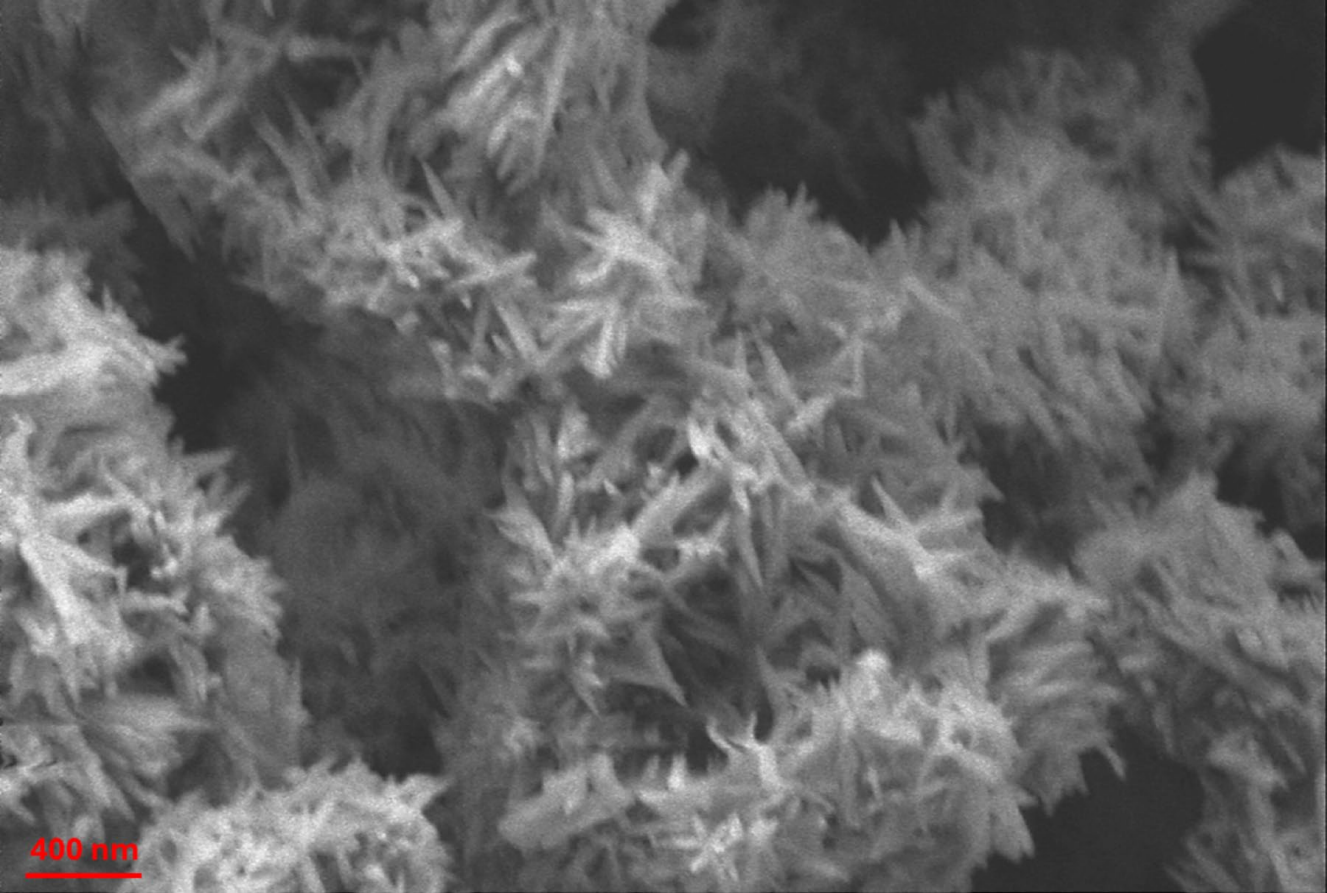
Scanning electron microscopy (SEM) images of the nano-calcite powder. **A**, **B** Morphological observations revealing porous, cauliflower-like secondary agglomerates composed of nanostructured calcite building blocks.

**Fig. 4:**
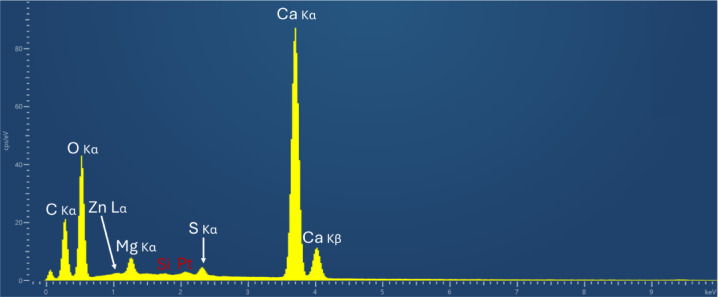
Energy-dispersive X-ray spectroscopy (EDS) spectrum of the nano-calcite powder, showing predominant Ca, O, and C peaks alongside trace signals of Mg, Zn, and S derived from the morphology-controlling additives.

Scanning electron microscopy (SEM) reveals that the macroscopic product consists predominantly of highly agglomerated, cauliflower-like particulate networks (Fig. 3), a morphology markedly different from the isolated rhombohedral crystals typically produced by uninhibited calcite precipitation. Energy-dispersive X-ray spectroscopy (EDS) further indicates a composition dominated by Ca and O, with trace Mg, Zn, and S signals reflecting the additive-mediated growth route (Fig. 4). This hierarchical aggregation is structurally compatible with the substantial pore volume and mesopore-dominated BJH distribution discussed in the section "Textural and porosity characteristics", and is consistent with an aggregation-mediated assembly of primary one-dimensional units during the later stages of carbonation^2^.

**Fig. 5:**
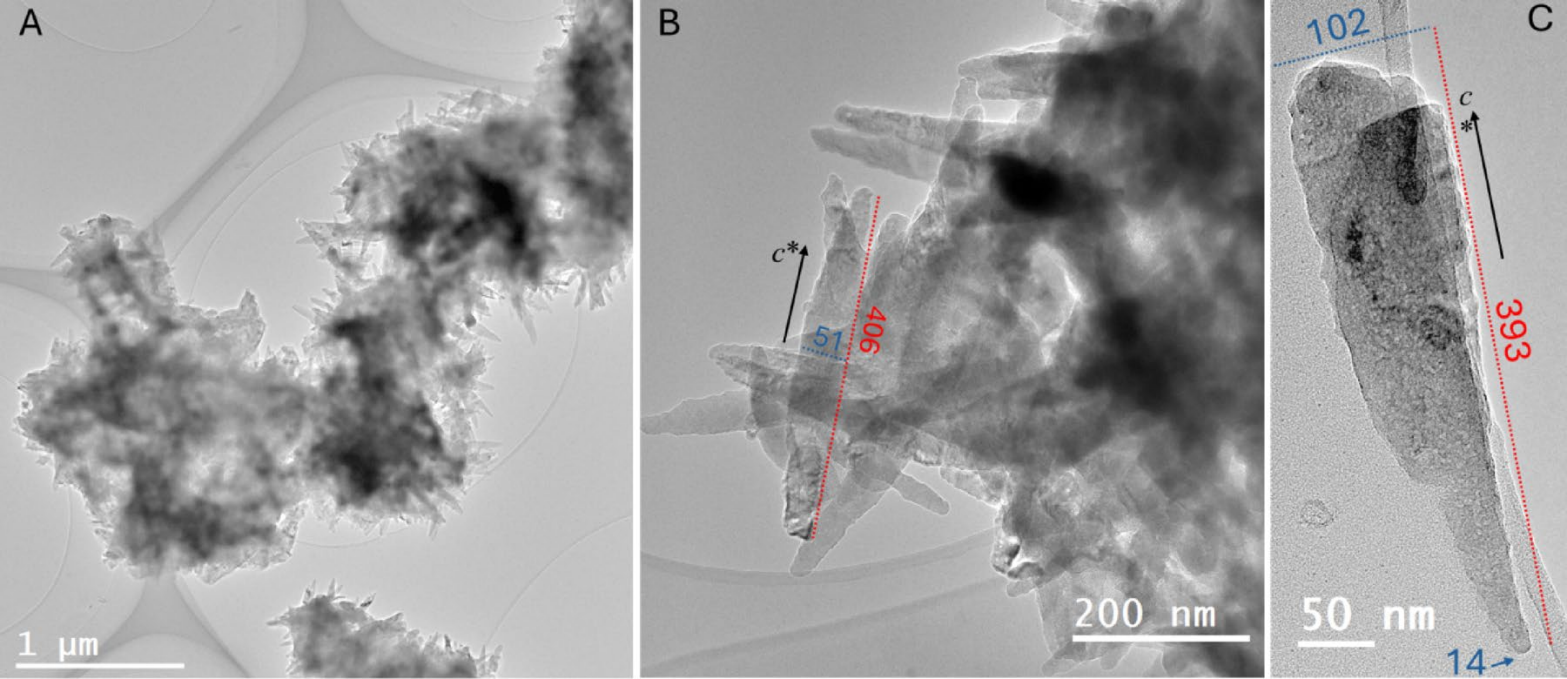
Transmission electron microscopy (TEM) images of the calcite nanorod/whisker building blocks. **A** Low-magnification image displaying the aggregated nano-units. **B**, **C** High-magnification images resolving the highly anisotropic, elongated rod/whisker-like particles. Representative dimensions are annotated directly on individual particles: red labels indicate the long-axis (length) measurement of each whisker (~ 393–406 nm), and blue labels indicate the corresponding lateral (width) measurement (~ 57–102 nm). Aspect ratios calculated from these annotated values fall in the range of 4–7.

**Fig. 6:**
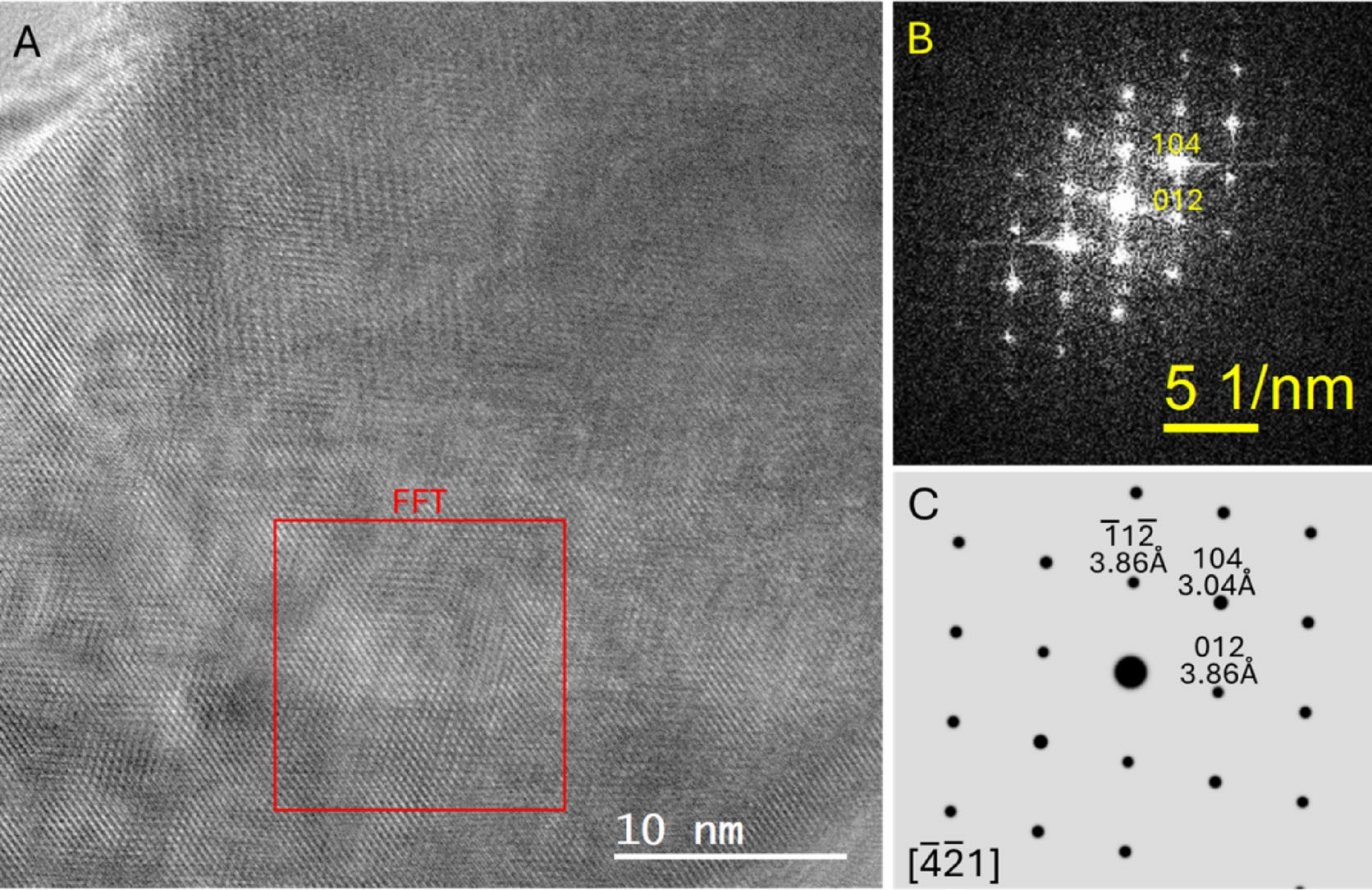
High-resolution TEM (HRTEM) and Fast Fourier Transform (FFT) analyses of the calcite lattice. **A** HRTEM image highlighting a well-ordered crystalline domain used for FFT analysis. **B** Corresponding FFT pattern exhibiting discrete, characteristic reflections. **C** Indexed diffraction-spot schematic detailing the representative *d*-spacings assigned to specific calcite crystallographic planes [(104) at *d* ≈ 3.04 Å and (012) at *d* ≈ 3.86 Å].

To resolve the internal architecture of these micron-scale aggregates and to identify the primary building blocks from which they are assembled, we next examined individual nano-units by transmission electron microscopy (TEM). TEM reveals that the aggregates are composed of highly anisotropic primary nano-units (Fig. 5A). At higher magnification, elongated nanorod- and whisker-like particles are clearly resolved, with lengths of approximately 393–406 nm and lateral widths of 57–102 nm, corresponding to aspect ratios in the range of 4–7 (Fig. 5B, C). This pronounced anisotropy stands in marked contrast to the near-equant rhombohedral morphologies produced under conventional supersaturation conditions in the absence of additives, where the six symmetrically equivalent {104} faces grow at uniform rates and yield aspect ratios close to unity^4,21^. High-resolution TEM imaging of individual whiskers resolves well-ordered lattice fringes, and the corresponding FFT patterns yield discrete reflections indexed to the calcite (104) (*d* ≈ 3.04 Å) and (012) (*d* ≈ 3.86 Å) planes (Fig. 6), confirming that the elongated nano-units are phase-pure, single-crystalline calcite at the local scale and complementing the bulk-averaged XRD identification of section.

We emphasize that the aspect-ratio data, in isolation, do not constitute direct crystallographic evidence for elongation along a specific axis: an aspect ratio of 4–7 is consistent with growth along multiple plausible crystallographic directions and could in principle also reflect defect-mediated growth. The crystallographic interpretation of the elongation direction is therefore deferred to section "Proposed facet-selective growth mechanism" (mechanism), where the HRTEM/FFT indexing established in the section "Hierarchical morphology across length scales" is integrated with the established literature on additive-mediated facet-selective growth. The morphological observations reported here are consistent with additive-mediated shape control, in which selective stabilization of specific facets by foreign ionic species redirects crystal habit toward rod-like morphologies—an effect well documented for calcite in the presence of Mg²⁺ and other inorganic and biomolecular additives^4,5,11,21,22^. We note that we did not collect direct evidence (for example, in situ tracking of pre-nucleation clusters, characterization of an ACC precursor, or imaging of particle-attachment events) for any specific nonclassical crystallization route in our system; we therefore refrain from assigning the observed pathway to that family of mechanisms. Direct identification of the elongation direction at the single-whisker level—for example, through systematic SAED indexing along multiple zone axes correlated with the long-axis vector of individual whiskers, or through electron tomography combined with crystallographic indexing—remains a valuable target for follow-up investigation.

### Textural and porosity characteristics

Nitrogen physisorption at 77 K yields a Brunauer–Emmett–Teller (BET) specific surface area of 9.58 ± 0.10 m² g⁻¹ for the synthesized nano-calcite powder, with a total pore volume of 0.131 cm³ g⁻¹ evaluated near the saturation pressure. Barrett–Joyner–Halenda (BJH) analysis reveals a prominent mesopore population centered at approximately 49.3 nm, indicating that the accessible porosity is governed predominantly by interparticle voids—capillary spaces between entangled nanowhiskers—and the complex packing geometry of the secondary agglomerates, rather than by intrinsic intraparticle microporosity. The measured BET surface area falls in an intermediate regime relative to typical commercial CaCO₃ products: it is substantially larger than that of ground calcium carbonate (GCC), which generally lies in the range of ~ 0.5–5 m² g⁻¹, and is broadly comparable to that of fine precipitated calcium carbonate (PCC) and biogenic high-aspect-ratio CaCO₃ products, which typically span ~ 5–25 m² g⁻¹ depending on particle size and morphology^2^. These textural characteristics place the synthesized material in an intermediate regime between dense, coarse calcite rhombohedra and highly dispersed nanoparticulates, consistent with a hierarchically agglomerated, permeable assembly of one-dimensional nanoscale building blocks^23,24^.

### Compositional signatures of additive interaction

Energy-dispersive X-ray spectroscopy (EDS) confirms a composition dominated by Ca and O, in agreement with the calcite phase identified by XRD (Fig. 4). Weak signals corresponding to Mg, S, and Zn are also detected at trace levels. The presence of these elements is consistent with residual incorporation or preferential surface adsorption of the MgSO₄ and ZnSO₄ additives employed during synthesis, serving as chemical fingerprints of the additive-mediated growth modification. The location and incorporation depth of these residual Mg, S, and Zn signals are further constrained by the structural analyses presented in the section "Hierarchical morphology across length scales": the absence of detectable peak shifts in bulk-averaged XRD, together with the absence of measurable lattice distortion in locally probed HRTEM/FFT, jointly argues against extensive substitution of these cations into the bulk calcite lattice. Taken together, these observations point to a surface-localized distribution of the additive-derived species. We note, however, that the present qualitative EDS dataset does not by itself distinguish between (i) chemisorbed surface species, (ii) loosely associated residual sulfate salts, and (iii) trace lattice-incorporated ions.

### Proposed facet-selective growth mechanism

**Fig. 7:**
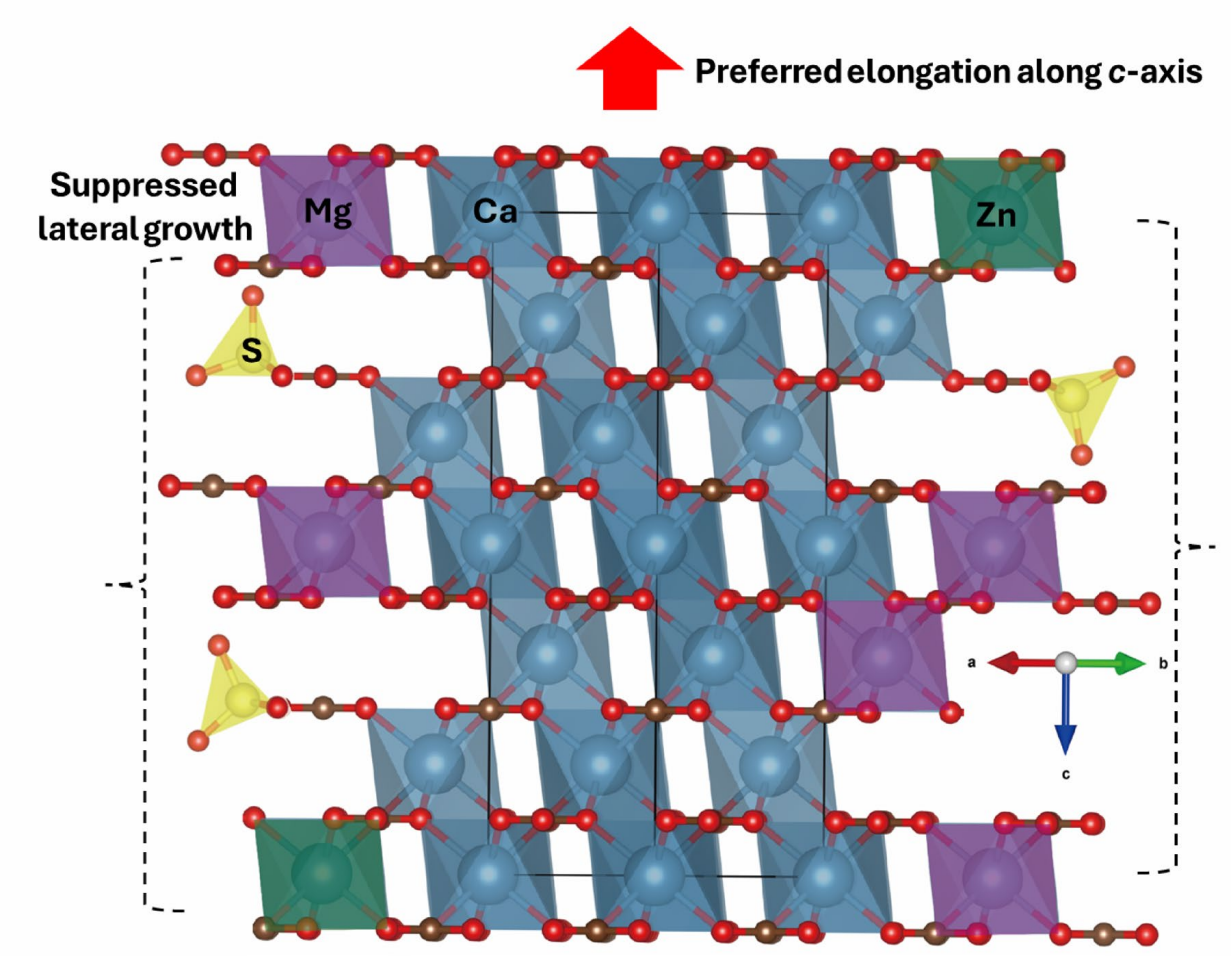
Schematic growth model illustrating the anisotropic formation of calcite whiskers. The conceptual mechanism highlights the proposed suppression of lateral growth along the *a*- and *b*-axes and the inferred preferential elongation along the crystallographic *c*-axis, driven by Mg/Zn/SO₄ additive-mediated, facet-selective interactions.

On the basis of the multiscale characterization data presented above, we propose a facet-selective, additive-assisted growth model for the formation of one-dimensional calcite nanowhiskers (Fig. 7). The choice of Mg²⁺, Zn²⁺, and SO₄²⁻ as co-additives is rooted in well-established crystal-chemical principles. Mg²⁺ (ionic radius ~ 0.72 Å) is substantially smaller than Ca²⁺ (~ 1.00 Å) and is among the most strongly hydrated divalent cations, with a high hydration enthalpy that imposes a kinetic barrier to its incorporation into the calcite lattice^11^. Geometric and electrostatic compatibility nonetheless allow Mg²⁺ to adsorb preferentially onto acute step edges of {104} faces, where it acts as an effective impurity poison^11,16^. Zn²⁺ (~ 0.74 Å), although similar in radius to Mg²⁺, is a softer cation that forms inner-sphere surface complexes with carbonate-bearing surfaces and tends to interact selectively with structurally distinct, more polar termination sites of carbonate minerals^13,14^. Sulfate (SO₄²⁻), while not incorporating substantially into the calcite lattice, perturbs the interfacial water structure and the local hydration of growth sites and modifies the relative free energies of crystallographically distinct calcite faces^6,12^. The selection of these three co-additives is therefore designed to combine, within a single staged carbonation route, a direct step-poisoning agent (Mg²⁺), a facet-selective surface-complexing agent (Zn²⁺), and an interfacial-water modifier (SO₄²⁻).

During the initial stages of carbonation, the pre-addition of MgSO₄ introduces effective impurity poisoning at reactive step and kink sites on nascent calcite surfaces^11,25^. According to classical step-pinning theory (the Cabrera–Vermilyea model)^25^, Mg²⁺ ions adsorb preferentially onto the acute step edges of {104} facets, driven by their high hydration enthalpy and geometric compatibility with the calcite surface structure, thereby retarding step propagation and suppressing lateral thickening along the *a*-axes^11^. This inhibitory effect is synergistically reinforced by sulfate species, which disrupt local hydration structures and modify the interfacial free energy^6,12^. In the subsequent stage, the continuous introduction of ZnSO₄ sustains the active surface state and provides additional facet-selective interactions through the formation of zinc-bearing surface complexes that bind preferentially to structurally distinct terminating facets^13,14^. Within this model, the combined kinetic inhibition along the lateral directions biases crystal development toward preferential mass deposition and anisotropic elongation along the crystallographic *c*-axis^16,22^. The proposed *c*-axis-selective elongation is consistent with (i) the highly anisotropic nano-unit morphology directly resolved by TEM, (ii) the crystallographic identity of individual whiskers as phase-pure, surface-modified calcite established jointly by XRD and HRTEM/FFT, and (iii) prior reports of *c*-axis-selective Mg²⁺ binding on biomimetic calcite surfaces^22,26^. We emphasize that direct experimental verification of the elongation direction at the single-whisker level (e.g., through SAED indexing along multiple zone axes correlated with the long-axis vector of individual whiskers) would be required to convert this proposed assignment into a directly demonstrated one; we identify this as a worthwhile target for follow-up studies.

The optimal additive dosage windows reported in the section "Synthesis procedure" (MgSO₄ at 0.01–0.03 mol per mol Ca(OH)₂, and ZnSO₄ at 0.02–0.15 mol per mol Ca(OH)₂) reflect a kinetic compromise that is qualitatively consistent with this mechanistic picture. At dosages substantially below the lower bound, the surface coverage of Mg²⁺ and Zn²⁺ is expected to be insufficient to suppress lateral step advancement, and the system relaxes toward the equilibrium rhombohedral habit dictated by the {104} faces. At dosages substantially above the upper bound, the high free Mg²⁺ activity is expected to drive the formation of Mg²⁺-rich amorphous calcium carbonate (Mg-ACC) intermediates whose subsequent transformation tends to favor aragonite or high-Mg calcite rather than the low-Mg, *c*-axis-elongated calcite reported here, in line with prior reports on Mg-mediated polymorph selection^9,10^. A systematic concentration-dependent study mapping the morphology, polymorph, and aspect-ratio distributions across the full additive composition space would directly verify these qualitative arguments; such a phase-space study, including independently varied MgSO₄, ZnSO₄, and H₂SO₄ dosages and extension to elevated temperatures and Mg²⁺/Ca²⁺ ratios, is identified as a focus of subsequent investigation. Within the dosage windows employed in the present work, the synergistic Mg/Zn/SO₄ additive system reproducibly preserves the thermodynamically stable calcite phase while redirecting its macroscopic habit toward mechanically robust one-dimensional whisker morphologies.

## Conclusions

In this study, we synthesized hierarchically nano-textured CaCO₃ comprising highly anisotropic calcite nanorods and whiskers via a staged Mg/Zn/SO₄ additive-assisted carbonation route. Phase and thermal analyses by XRD and TG/DTG, complemented by atomic-resolution HRTEM/FFT, confirmed the preservation of the thermodynamically stable calcite phase with high compositional purity, with the convergence of bulk- and local-scale structural evidence indicating that the additive-derived Mg^2+^, Zn^2+^, and SO_4_^2-^ species reside predominantly at crystal surfaces rather than within the bulk lattice. Textural and morphological characterization by N₂ physisorption, SEM, and TEM demonstrated that these sub-micrometer one-dimensional units (approximately 393–406 nm in length) assemble into highly porous secondary agglomerates featuring an extensive mesoporous network and an intermediate specific surface area. On the basis of these multiscale observations, we propose a facet-selective crystallization mechanism in which synergistic kinetic inhibition by Mg²⁺ and SO₄²⁻ suppresses lateral thickening along the *a*- and *b*-axes, while Zn^2+^-mediated surface interactions are inferred to promote anisotropic elongation along the crystallographic *c*-axis. Direct crystallographic verification of the elongation direction at the single-whisker level, quantitative partitioning of the additive-derived signal between surface and bulk reservoirs (e.g., through XPS and ICP analyses of washed specimens), quantitative whisker-induced texture analysis (e.g., Rietveld refinement with preferred-orientation correction), and systematic mapping of the additive-composition–morphology phase space remain natural extensions of the present study and are explicitly identified as foci for future work. Within these clearly stated boundaries, the present findings offer mechanistic insights into additive-directed morphology control during mineral carbonation and a scalable pathway for engineering one-dimensional calcite architectures applicable to advanced structural composites and environmental technologies.

## Data Availability

For access to the measured experimental data, you can reach Seungyeol Lee via email at slee2@cbnu.ac.kr.
